# HEPATOSPLENIC SCHISTOSOMIASIS-ASSOCIATED CHRONIC PORTAL VEIN THROMBOSIS: RISK FACTOR FOR HEPATOCELLULAR CARCINOMA?

**DOI:** 10.1590/0102-672020230045e1763

**Published:** 2023-09-15

**Authors:** George Felipe Bezerra Darce, Fabio Ferrari Makdissi, Sabrina de Mello Ando, Gilton Marques Fonseca, Jaime Arthur Pirola Kruger, Fabricio Ferreira Coelho, Manoel de Souza Rocha, Paulo Herman

**Affiliations:** 1 Universidade de São Paulo, Falculty of Medicine, Department of Gastroenterology – São Paulo (SP), Brazil;; 2 Universidade de São Paulo, Faculty of Medicine, Cancer Institute – São Paulo (SP), Brazil;; 3 Universidade de São Paulo, Falculty of Medicine, Department of Radiology –São Paulo (SP), Brazil.

**Keywords:** Schistosomiasis, Portal vein, Thrombosis, Hepatocellular carcinoma, Esquistossomose, Veia porta, Trombose, Carcinoma hepatocellular

## Abstract

**BACKGROUND:**

Hepatosplenic schistosomiasis is an endemic disease prevalent in tropical countries and is associated with a high incidence of portal vein thrombosis. Inflammatory changes caused by both parasitic infection and portal thrombosis can lead to the development of chronic liver disease with potential carcinogenesis.

**AIMS:**

To assess the incidence of portal vein thrombosis and hepatocellular carcinoma in patients with schistosomiasis during long-term follow-up.

**METHODS:**

A retrospective study was conducted involving patients with schistosomiasis followed up at our institution between 1990 and 2021.

**RESULTS:**

A total of 126 patients with schistosomiasis were evaluated in the study. The mean follow-up time was 16 years (range 5–31). Of the total, 73 (57.9%) patients presented portal vein thrombosis during follow-up. Six (8.1%) of them were diagnosed with hepatocellular carcinoma, all with portal vein thrombosis diagnosed more than ten years before.

**CONCLUSIONS:**

The incidence of hepatocellular carcinoma in patients with schistosomiasis and chronic portal vein thrombosis highlights the importance of a systematic long-term follow-up in this group of patients.

## RESUMO

**RACIONAL:**

A esquistossomose hepatoesplênica é uma doença endêmica prevalente em países tropicais e está associada a uma alta incidência de trombose da veia porta. Alterações inflamatórias causadas tanto pela infecção parasitária quanto pela trombose portal podem levar ao desenvolvimento de doença hepática crônica com potencial carcinogênico.

**OBJETIVOS:**

Avaliar a incidência de trombose da veia porta e carcinoma hepatocelular em pacientes com esquistossomose durante um seguimento de longo prazo.

**MÉTODOS:**

Foi realizado estudo retrospectivo envolvendo pacientes com esquistossomose acompanhados em nossa instituição entre 1990 e 2021.

**RESULTADOS:**

Um total de 126 pacientes com esquistossomose foram avaliados no estudo. O tempo médio de acompanhamento foi de 16 anos (variando de 5 a 31). Do total, 73 (57,9%) pacientes apresentaram trombose da veia porta durante o seguimento e seis (8,1%) deles foram diagnosticados com carcinoma hepatocelular, todos com trombose da veia porta diagnosticada há mais de 10 anos.

**CONCLUSÕES:**

A incidência de carcinoma hepatocelular em pacientes com esquistossomose e trombose da veia porta crônica destaca a importância de um acompanhamento sistemático de longo prazo nesse grupo de pacientes.

## INTRODUCTION

Schistosomiasis is a chronic disease that infects approximately 240 million people worldwide and nearly 700 million individuals live in endemic areas^28^. The infection is prevalent in tropical and subtropical poor communities where adequate sanitation is lacking.

The hepatosplenic form of the disease, also known as hepatosplenic schistosomiasis (HSS) or schistosomal portal hypertension, is characterized by the presence of periportal liver fibrosis, with presinusoidal portal hypertension, formation of collateral circulation, and the development of esophageal and/or gastric varices without liver dysfunction^3,18^. The presentation of the disease is associated with chronic infection, high parasite load, and/or reinfection with *S. mansoni*. The incidence of HSS is approximately 5–10%, presenting high morbidity and mortality, mainly owing to the risk of gastrointestinal (GI) bleeding caused by the rupture of the esophageal or gastric varices^22^. The main treatment for secondary prevention of GI bleeding in patients with HSS is esophagogastric devascularization and splenectomy (EGDS)^10^, and the most frequent complication is portal vein thrombosis (PVT)^19^.

The reported incidence of PVT in HSS patients is 33%^6^. A high incidence ranging from 17–63% is observed in patients undergoing splenectomy^4,6,9,19^, with a 6.12-fold higher risk^4^. PVT can further accentuate portal hypertension with complications, such as GI bleeding due to varices, ascites, portal biliopathy, and portosystemic encephalopathy^16^. Moreover, it is associated with a hepatic carcinogenic potential since reduced hepatic blood supply causes parenchymal changes^14^.

In addition to PVT, schistosomiasis itself seems to be associated with hepatic carcinogenic potential^1,23,25,28^. Both experimental and clinical studies in the last 40 years sought to demonstrate a direct association between schistosomiasis and primary liver cancer^12^. However, this relationship is still poorly established^28^. Although schistosomiasis and PVT are related to the pathogenesis of hepatocellular carcinoma (HCC), no studies on the occurrence of HCC in patients with both schistosomiasis and PVT are available.

This study aimed to assess HCC incidence in patients with HSS and its association with PVT during long-term follow-up.

## METHODS

This retrospective study included patients from a single referral center. Schistosomiasis was diagnosed based on epidemiological, clinical, and laboratory data and further confirmed by histopathological evaluation.

The diagnosis of the hepatosplenic form of the disease was based on the presence of splenomegaly, esophageal varices, and periportal fibrosis on liver ultrasound evaluation. Patients with previous episodes of upper digestive bleeding from esophageal varices rupture underwent EGDS as a treatment for portal hypertension. All patients followed up at our institution between 1990 and 2021 were enrolled in our database. Patients with a history of alcoholism, nonalcoholic steatohepatitis, hepatitis B or C viral infection were excluded.

The patients were analyzed for different parameters, including sex, age, follow-up time, presence of comorbidities and PVT, surgery for portal hypertension treatment, diagnosis, time for diagnosis, and treatment for HCC.

**Figure 1:**
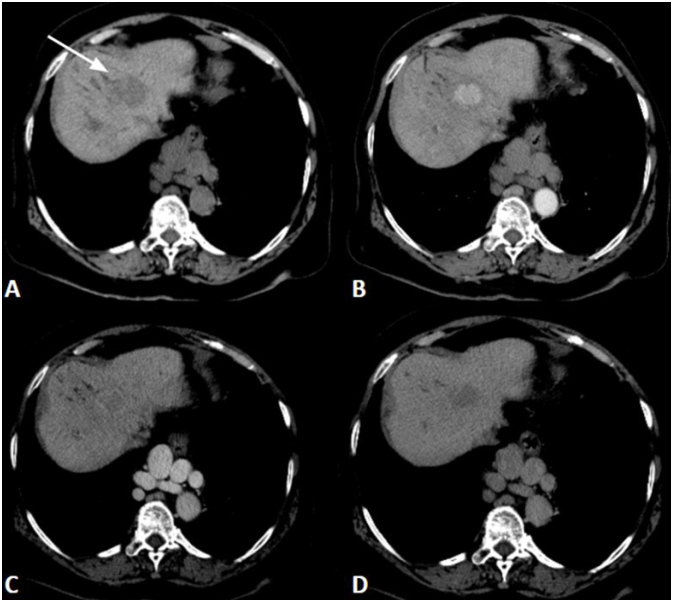
Hepatocellular carcinoma in a 68-year-old woman with hepatosplenic schistosomiasis. A: Unenhanced axial computed tomography image showing hypoattenuating nodule (arrow); B: Hypervascularized nodule in arterial phase; C and D: Washout in delayed phase.

PVT was diagnosed using Doppler ultrasonography of the portal system, performed annually during patient follow-up, according to our institutional protocol. HCC was first diagnosed by routine annual ultrasound and confirmed using axial imaging, abdominal computed tomography, or magnetic resonance imaging with intravenous contrast, according to the recommendations of the American Association for the Study of Liver Diseases (AASLD)^16^ as illustrated in Figure 1.

Statistical analyses were performed using JASP 0.14.1 statistical software (University of Amsterdam). Categorical variables were compared through Pearson's chi-square (χ^2^) test and continuous variables were assessed by Student's *t*-test. Two-tailed p-values<0.05 were considered statistically significant. The study was approved by the Ethics Committee for the Analysis of Research Projects of the Institution (nº 61364522.6.0000.0068).

## RESULTS

A total of 126 patients monitored during the study period were evaluated. The mean follow-up time was 16 years (range 5–31). Of all patients, 71 (56.3%) were female, and the mean age was 57 years (range 35–83). During the late follow-up, 73 (57.9%) patients presented PVT.

The characteristics of patients with and without PVT are summarized in Table 1. No significant differences were found between these two groups regarding sex, age, and follow-up time. PVT was more frequent in patients who underwent EGDS (65.7 vs. 34.0%, p<0.001).

**Table 1:** Characteristics of patients with hepatosplenic schistosomiasis based on the presence of portal vein thrombosis.

		With PVT	Without PVT	p
Total		73	53	
Age, mean (years)		58	57	0.189
Sex (%)				
	Female	39 (53.4)	32 (60.3	0.402
	Male	34 (46.6)	21 (39.7)	
	Follow-up time (years)	16	16	0.459
EGDS (%)				
	Yes	48 (65.7)	18 (34.0)	<0.001
	No	25 (34.3)	35 (66.0)	
	HCC	6 (8.1)	0	0.032

PVT: portal vein thrombosis; p: p-value; EGDS: esophagogastric devascularization and splenectomy; HCC: hepatocellular carcinoma.

Among PVT patients, six (8.1%) were diagnosed with HCC. HCC was not found in patients without PVT (p=0.032). All patients with HCC had the diagnosis of PVT more than ten years before. The characteristics of patients with HCC are summarized in Table 2.

**Table 2:** Patients with hepatosplenic schistosomiasis and portal vein thrombosis diagnosed with hepatocellular carcinoma.

Case	Sex	Age (years) at diagnosis of HCC	Follow-up time (years)	Time (years) between PVT and HCC	Alpha-fetoprotein (ng/dL)	Treatment
Case 1	M	49	23	23	1888.0	Palliative
Case 2	F	68	17	17	12321.0	Palliative
Case 3	F	68	17	10	2.7	LT
Case 4	F	61	29	15	10.1	SBRT
Case 5	M	53	28	10	1.9	LT
Case 6	F	64	31	25	2.3	SBRT

HCC: hepatocellular carcinoma; PVT: portal vein thrombosis; M: male; F: female; LT: liver transplant; SBRT; stereotactic body radiotherapy.

Two patients diagnosed with HCC could not be treated with curative intent because they were presented with decompensated liver disease at the diagnosis, and both died shortly after. Two patients with contraindications for surgical or ablative treatment were referred to stereotactic body radiotherapy. Another two underwent arterial chemoembolization of the tumor and, thereafter, liver transplantation^11,12^.

## DISCUSSION

The association between HSS and HCC has not been clearly established^7,25,26,27^. Schistosomiasis could be associated with carcinogenesis due to different mechanisms such as chronic inflammation, affecting the production of proteins and modulation of enzymatic activities and gene expression^23^.

Previous studies that found an association between HSS and HCC were biased by the presence of patients with other chronic liver diseases, such as hepatitis B and C infections^8^. A study conducted in Egypt reported that 61.3% of patients with concomitant hepatitis C and schistosomiasis infection developed HCC compared with 38.7% of patients with HCC without schistosomiasis, suggesting that this co-infection significantly increased the incidence of HCC^7^. Another study at our institution — a case series, reported seven patients with schistosomiasis infection and HCC, but 57.1% had hepatitis B virus infection^27^. Our study focused on a group of patients with pure schistosomiasis excluding those with other associated liver diseases, with no overlap with the previous study.

A study that evaluated the molecular mechanisms linking *S. mansoni* infection and HCC showed that substances released from parasite eggs in tissues trigger the permanent activation of the c-Jun and STAT3 proto-oncogenes contributing to the development of HCC^5^. Histological analysis has shown dysregulation of fibrosis formation in hepatocytes of patients with schistosomiasis, related to mutations in the PIK3CA and TP53 genes^1^. Changes in these genes can also be related to the development of HCC^1^.

Experimental studies on PVT also suggested its association with HCC development. Studies using portal vein ligation in rats demonstrated a long-term effect of portal flow deprivation on mRNA expression of proliferative and angiogenic factors in the liver, indicating a potential association with liver carcinogenesis^13,14^. Another study showed an association of PVT with the development of liver lesions, including benign liver tumors, such as focal nodular hyperplasia and hepatocellular adenoma, and malignant liver tumors, such as HCC and hepatoblastoma^15,17^.

Schistosomiasis is associated with a higher incidence of PVT in up to 33% of patients^4^. Potential mechanisms are vascular alterations and endothelial injury in presinusoidal medium-sized portal system branches due to periportal fibrosis and portal hypertension^3,20^. An even more significant incidence of PVT is observed in patients who underwent EGDS, which is considered the most frequent complication of this surgery, with an incidence of 63%^18^. Despite this high incidence, there is no consensus on the follow-up regimen or tracking of postoperative PVT^11,12^.

The diagnosis of HCC can be made exclusively through imaging exams, unlike other neoplasms. The aspects of the image are hypervascularization in the arterial phase and washout in the late phases. These characteristics are standardized by LIRADS (Liver Imaging Reporting and Data System), which was created to standard reports, categorizing lesions into probabilities of being HCC, ranging from 1 (definitely benign lesion) to 5 (definitely HCC)^21^.

We can use these typical characteristics to evaluate liver nodules in patients with schistosomiasis, but in cirrhosis, due to a vascular disorder such as the chronic portal vein occlusion, LIRADS is not applicable^2^. Imaging predictive value for HCC may not be sufficiently high in such patients^2^.

Thus, depending on the case, other methods should be employed to investigate those lesions, such as hepatospecific contrasts or even complementation with histopathological examination^24^. In our study, HCC cases had typical image characteristics, and in two patients, HCC presented portal vein tumoral thrombosis.

The present study has limitations since it is retrospective with a small sample size, thereby precluding comparative analyses with statistical power. The study highlights an 8.1% incidence of HCC in patients with schistosomiasis and chronic PVT, and also emphasizes the absence of HCC in those patients without PVT during the long-term follow-up. Patients with HSS are followed for a long time in our institution, regardless of the presence or absence of PVT. This includes an annual evaluation with liver function tests, abdominal ultrasound portal system Doppler, and upper GI endoscopy. However, only two of the six patients diagnosed with HCC underwent curative treatment, indicating that the diagnosis may occur in an advanced cancer stage if there is not adequate follow-up. Thus, a careful clinical and radiological long-term follow-up is needed for HSS patients with PVT, especially those following EGDS.

## CONCLUSIONS

Our study highlights the incidence of HCC in patients with schistosomiasis and PVT. The presence of PVT, a frequent finding in HSS patients, especially in those who underwent EGDS, after long-term follow-up may represent a risk for the development of HCC. Based on our findings, we suggest that such patients should undergo a systematic long-term follow-up (over 10 years) using imaging tests to screen for HCC.
